# Modulation of inflammatory and adrenergic pathways in hypertension: effects of β-blockers on cytokine release in Jurkat T cells

**DOI:** 10.3389/abp.2025.14935

**Published:** 2025-10-17

**Authors:** Nana Kajaia, Maia Enukidze, Marine Machavariani, Tinatin Maminaishvili, Sophio Kalmakhelidze, George Ormotsadze, Tamar Sanikidze

**Affiliations:** ^1^ Department of Physics, Biophysics, Biomechanics and Informative Technologies of Tbilisi State Medical University, Tbilisi, Georgia; ^2^ Bakhutashvili Institute of Medical Biotekhnology of Tbilisi State Medical University, Tbilisi, Georgia; ^3^ School of Dental Medicine of BAU International University, Batumi, Georgia

**Keywords:** hypertension, β-blockers, cytokines, Jurkat T cells, adrenergic pathways

## Abstract

Research aimed to examine the effects of β-blockers on cytokine release in Jurkat cells under basal conditions and during oxidative stress. Oxidative stress was induced in Jurkat cells through the application of hydrogen peroxide (H_2_O_2_). Subsequently, β-blockers were administered to the incubation medium for 24 h, encompassing both intact and oxidatively stressed cell conditions. For β-blocker toxicity screening, the viability of Jurkat cells was determined using the MTT (3-(4,5-dimethylthiazolyl-2)-2,5-diphenyltetrazolium bromide) test. The IL-6, IL-17, and TNF-α content were measured in the supernatant of Jurkat cells incubated under different conditions. The study results show that propranolol, metoprolol, carvedilol, but not nebivolol, revealed toxic effects on the intact Jurkat cells (p_c-p_ = 0.0001; p_c-m_ > 0.0001; p_c-c_ = 0.0003; p_c-n_ = 0.0525). Under oxidative stress conditions, the viability of Jurkat cells decreased significantly (p_c-H2O2_ = 0.0001). Propranolol and metoprolol did not affect ((p_c-p_ = 0.0001; p_c-m_ > 0.0001), while nebivolol and carvedilol improved the viability of Jurkat cells incubated under oxidative stress conditions (p_c-n_ = 0.002; p_c-c_ = 0.0002). Oxidative stress significantly increased the cytokines (IL-6, TNF-α, IL-17) expression levels (p_c-H2O2_ < 0.0001; p_c-H2O2_ < 0.0001; p_c-H2O2_ < 0.0001) in Jurkat cells. Propranolol, carvedilol, nebivolol, and metoprolol did not significantly affect the expression levels of IL-6, TNF-α, and IL-17 in intact Jurkat cells, but decreased IL-6, TNF-α, and did not change IL-17 expression levels in Jurkat cells incubated under oxidative stress conditions. This study demonstrates that β-blockers can influence redox-sensitive cytokine pathways in Jurkat T lymphocytes when they are under oxidative stress. All the agents tested inhibited the production of IL-6 and TNF-α, but nebivolol and carvedilol showed the strongest protective and anti-inflammatory effects. These effects likely result from their combined properties, including antioxidant effects, nitric oxide modulation, and the regulation of NF-κB/MAPK pathways. In contrast, propranolol and metoprolol exhibited more limited activity. These findings suggest that third-generation β-blockers may offer both cardiovascular and immunomodulatory benefits, although further validation in primary immune cells and *in vivo* models is still required.

## Introduction

Hypertension is a significant contributor to the global burden of cardiovascular disease, leading to considerable morbidity and mortality worldwide. It affects over a billion people and is recognized as a primary modifiable risk factor for serious conditions such as stroke, myocardial infarction, heart failure, and chronic kidney disease (Chobanian et al., 2003). The pathogenesis of essential arterial hypertension is highly complex and multifactorial. Despite nearly a century of research, finding effective treatment strategies continues to be a major challenge in clinical medicine.

The traditional understanding of hypertension focused primarily on issues related to hemodynamic regulation and fluid balance, which were thought to be driven mainly by changes in vascular resistance, renal sodium handling, and neurohormonal regulation. However, recent evidence from the past two decades suggests that immune and inflammatory mechanisms play significant roles in its pathophysiology. Specifically, the interaction between chronic low-grade inflammation and the activity of the adrenergic system has become a crucial factor in both the onset and progression of hypertensive disease (Harrison et al., 2011; Madhur et al., 2010).

A growing body of experimental and clinical research has identified inflammation as a key mechanism in initiating and maintaining elevated blood pressure. Inflammation contributes to endothelial dysfunction, vascular remodelling, and increased arterial stiffness. These effects are mediated by pro-inflammatory cytokines, including tumour necrosis factor-alpha (TNF-α), interleukin-6 (IL-6), interleukin-17 (IL-17), and interleukin-1β (IL-1β), as well as chemokines such as monocyte chemoattractant protein-1 (MCP-1). These mediators facilitate immune-cell recruitment and activation within the vascular wall, leading to endothelial impairment, tissue injury, and renal dysfunction - all of which contribute to hypertensive pathology (Sriramula et al., 2008; Zhang et al., 2012; Dinh et al., 2014; Li et al., 2025). Inflammatory mediators also activate the renin-angiotensin-aldosterone system (RAAS), thereby contributing to blood pressure elevation and end-organ damage (Wenzel et al., 2011). Targeting these cytokines may therefore provide novel therapeutic strategies, particularly in treatment-resistant hypertension.

Chronic overactivation of the central nervous system (CNS), a hallmark of many forms of hypertension, not only sustains elevated blood pressure but also amplifies inflammatory signalling. Recent studies have demonstrated a link between inflammation and sympathetic activation in hypertension development, mediated through α- and β-adrenergic receptors (α1, α2, β1, β2) expressed on immune cells, including T cells. These receptors influence T-cell activation and cytokine production and promote leukocyte adhesion, migration, infiltration, and apoptosis (Wenzel et al., 2011). Experimental models have shown that T-cell depletion or cytokine blockade can attenuate hypertension induced by angiotensin II or norepinephrine infusion, highlighting the importance of immune effectors in mediating hypertensive responses (Guzik et al., 2007). Likewise, pharmacological or surgical inhibition of sympathetic nerve activity reduces cytokine expression and immune-cell infiltration in hypertensive organs (Marvar et al., 2010). This bidirectional crosstalk creates a self-amplifying loop in which inflammation and sympathetic activation perpetuate one another, sustaining hypertension and promoting organ damage. Understanding these mechanisms is essential for designing immune-modulatory approaches to hypertension.

β-adrenergic blockers (β-blockers) are a well-established class of antihypertensive drugs that act by antagonising β-adrenergic receptors. Beyond their hemodynamic actions, β-blockers modulate immune activity - reducing pro-inflammatory cytokine production, including TNF-α and IL-6, while enhancing anti-inflammatory cytokines such as IL-10 (Gaskill and Khoshbouei, 2022). They also mitigate inflammatory responses implicated in endothelial dysfunction and vascular remodelling (Harrison et al., 2011). This dual action - modulating CNS activity and inflammatory pathways - positions β-blockers as both neurohormonal and immunomodulatory agents in hypertension treatment.

Based on pharmacological selectivity, β-blockers are classified into three generations: first-generation (non-selective, e.g., propranolol, which blocks both β_1_ and β_2_ receptors), second-generation (β_1_-selective, e.g., metoprolol), and third-generation (agents with vasodilatory and pleiotropic effects, e.g., nebivolol and carvedilol) (Frishman and Saunders, 2011). Nebivolol is characterised by high β_1_-selectivity at low doses and additional β_3_-adrenergic agonist activity, which promotes nitric oxide (NO) release, improves endothelial function, and confers antioxidant protection (Maffei and Lembo, 2009). Carvedilol, by contrast, combines non-selective β-blockade with α_1_-receptor antagonism, producing vasodilation and enhancing antioxidative and anti-inflammatory effects (Chen-Scarabelli et al., 2012).

Understanding the molecular mediators that connect inflammation and adrenergic receptor signalling in hypertension is essential for identifying novel therapeutic targets. This study aimed to examine the effects of β-blockers on cytokine release in Jurkat cells under basal conditions and during oxidative stress.

Jurkat cells, an immortalised human CD4^+^ T-cell line, preserve functional T-cell receptor (TCR)-mediated signalling and cytokine production. They are widely used in immunological research for studying T-cell survival, proliferation, differentiation, and activation (Szelényi and Vizi, 2007; Sharma and Farrar, 2020). Jurkat cells express a broad cytokine repertoire, including IL-2, IL-4, IL-6, IL-8, IL-10, IL-12, IL-17, TNF-α, interferon-γ (IFN-γ), and granulocyte-macrophage colony-stimulating factor (GM-CSF), triggered by diverse stimuli such as TCR signalling, mitogens (e.g., phorbol myristate acetate), superantigens (e.g., staphylococcal enterotoxins), cytokines (IL-2, IL-6, IL-12, IL-23, TNF-α), growth factors, pathogen-associated molecular patterns (PAMPs) (e.g., lipopolysaccharide), oxidative stress, and genetic manipulations, including transcription factor overexpression (e.g., retinoic acid receptor-related orphan receptor gamma, (RORγt) or CRISPR/Cas9-based gene editing (Suzuki et al., 2006; Kumar et al., 2014; Pawelec et al., 1982; Khalaf et al., 2010; Mori et al., 1996; Liu et al., 2005; Penix et al., 1993; Li-Weber and Krammer, 2003; Dinarello, 2000; Chen et al., 2024; Zhou and Littman, 2009; Li et al., 2023).

Jurkat cells predominantly express β_2_-adrenergic receptors, which makes them suitable for evaluating adrenergic modulation of T-cell function. Given that β-blockers exert pleiotropic effects by targeting β-adrenergic receptors on immune cells, Jurkat cells provide a mechanistically relevant platform for studying how β-blockers alter catecholamine-induced cytokine responses (Sanders, 2012).

## Materials and methods

### Cell culture and experimental design

Human leukemic mature T cells (Jurkat cells) (DSMZ- Deutsche Sammlung von Mikroorganismen und Zellkulturen (Germania)) were proliferated in bioactive medium RPMI 1640 (GIBCO), inactivated fetal bovine serum (Sigma), L-glutamine (4 mM), penicillin (100units/mL), and streptomycin (100units/mL) containing suspension. Cell culture was maintained at 37 °C in a humidified incubator with 5% CO_2_ and was passaged every 2–3 days to maintain exponential growth. Jurkat cells were seeded at a concentration of 0.3–0.6 × 10^6^ cells per millilitre of culture medium (RPMI-1640 supplemented with 10% fetal bovine serum and 1% penicillin-streptomycin). This cell density was chosen to ensure optimal viability and responsiveness during experimental treatments, while preventing overgrowth or nutrient depletion throughout the incubation period.

In designated experiments, oxidative stress (model of inflammatory conditions *in vitro*) was induced by the addition of hydrogen peroxide (H_2_O_2_; Sigma-Aldrich, USA) directly to the culture medium at concentrations of 50 µM (≈1.7 μg/mL; MW ≈ 34 g/mol) (typically used to study signalling pathways or antioxidant responses without inducing high cytotoxicity) for 24 h (Chiaramonte et al., 2001).

β-blockers were applied at therapeutic concentrations, prepared in physiological saline (0.9% NaCl), and added to the incubation medium of intact and oxidatively stressed Jurkat cells. Incubations were maintained for 24 h.

### Drug preparation and treatment

β-blockers (propranolol, carvedilol, nebivolol, and metoprolol) were selected for *in vitro* testing based on their clinical relevance. The daily therapeutic dose ranges in adults (assuming an average body weight of 70 kg) were taken from authoritative clinical sources (Mayo Clinic, Drugs.com) (Mayo Clinic, 2025a; Mayo Clinic, 2025b; Drugs.com, 2025; Mayo Clinic, 2025c; Mayo Clinic, 2025d).

For *in vitro* exposures, we employed concentration ranges (4–36 μg/mL) that are commonly used in cell culture experiments with β-blockers to ensure measurable biological responses (Hajatbeigi and Hajighasemi, 2018; Münzel and Gori, 2009; Fjæstad et al., 2022). These concentrations are not direct reflections of *in vivo* pharmacokinetic plasma levels, which are typically reported in the nanogram per millilitre (ng/mL) range (Houshyar et al., 2024; Li et al., 2022; Von et al., 1982). Instead, the selected concentrations should be interpreted as supra-physiological exposures suitable for mechanistic exploration *in vitro.*

Jurkat cells were seeded into 96-well plates at a density of 1 × 10^5^cells/well in 200 µL total volume. The β-blockers propranolol, carvedilol, nebivolol, and metoprolol (Sigma-Aldrich, USA) were prepared as aqueous stock solutions in complete culture medium. Because several β-blockers (notably carvedilol, propranolol) are poorly soluble in purely aqueous buffers near neutral pH, we prepared concentrated working stocks in the cells’ incubation medium (RPMI-1640), avoiding the use of DMSO or ethanol, which can alter Jurkat cell viability and oxidative stress markers. This ensured physiological osmolarity and pH, complete dissolution, and compatibility with serum-containing conditions. Using working stocks of 20–40 µL per 200 µL well (a - 20 μL, b - 40 µL) allowed for accurate pipetting of physiologically equivalent doses without exceeding solubility limits in serum-containing medium and minimised precipitation artefacts.

General dilution formula (per well):

$$C_{\text{stock}}=C_{\text{final}}\times \frac{V_{\text{final}\;\text{well}}}{V_{\text{stock}\;\text{added}}}$$

Where: C_final_ - target well concentration (36 μg/mL for propranolol); V_final, well_ = 200 μL; V_stock, added_ = 20 μL; 40 µL.

$$C_{\text{stock}}=36\;µg/\text{ml }\frac{200\;µl}{20\;µl}=360\;µg/\text{ml}$$

$$C_{\text{stock}}=36\;µg/\text{ml }\frac{200µl}{40\;µl}=180\;µg/\text{ml}$$

Prepared stock concentrations:

**Table:** 

Drug	Final conc. (µg/mL)	Stock (20 µL/well)	Stock (40 µL/well)
Propranolol	36	360 μg/mL	180 μg/mL
Carvedilol	10	100 μg/mL	50 μg/mL
Nebivolol	4	40 μg/mL	20 μg/mL
Metoprolol	20	200 μg/mL	100 μg/mL

The additions of 20–40 µL constituted 10%–20% of the total well volume; however, because the added volume consisted of the same incubation medium, the final nutrient and ionic composition remained consistent. Importantly, these additions did not alter the number of cells per well, as cells were seeded at a predetermined fixed density before treatment. Consequently, while the overall volume of the culture medium increased, the cell density (cells per ml) decreased proportionally. It is worth noting that this reduction in cell density was minimal and uniform across all wells within the same treatment group.

Two-volume approach allowed us to balance solubility stability, accurate dosing, and methodological consistency across compounds with different therapeutic-equivalent concentrations.

To screen for β-blocker toxicity, we evaluated the viability and proliferative activity of Jurkat cells using the MTT (3-(4,5-dimethylthiazol-2-yl)-2,5-diphenyltetrazolium bromide) assay. This method is a standard approach for initial cytotoxicity assessment due to its simplicity, reproducibility, and sensitivity (Berridge et al., 2005; Stockert et al., 2018). Since β-blockers directly affect mitochondrial function, including calcium homeostasis, membrane potential, and reactive oxygen species (ROS) generation (Debbasch et al., 2001; Nakamura et al., 2011; Shawgo et al., 2008), MTT reduction was used as a relevant endpoint reflecting both cell survival and mitochondrial activity.

### Cell viability assay

Cell suspensions (2 × 10^6^ cells/mL) were incubated with H_2_O_2_ and various β-blockers, as described above. After incubation, the cells were collected by centrifugation at 1500 × g for 5 min, washed once with phosphate-buffered saline (PBS), and resuspended in fresh culture medium. The 8 mg/mL solution of MTT (Sigma) in buffer (140 mM NaCl, 5 mM HEPES, pH 7.4) was added to the cell suspension at a rate of 30 μL per 100 μL suspension, and the mixture was incubated for 4 h at 37 °C in a humidified 5% CO_2_ atmosphere. After this incubation, the supernatant was carefully removed, and the formazan crystals produced from MTT were dissolved in 100 μL of dimethyl sulfoxide (DMSO). The absorbance values of the solutions were measured at a wavelength of 570 nm.

The absorbance (A) values obtained from experimental groups (A_sample_) were compared to those of the control (A_control_), and the viability coefficient (K) was expressed as a percentage according to the formula:

$$K=A_{\text{ sample}}\;/\;A_{\text{ control}}\times 100$$

where A_sample_ is the absorbance of the treated cells, and A_control_ is the absorbance of the untreated control group (representing 100% viability).

The primary aim of our study was to explore the immune-related effects of β-blockers on T-lymphocytes. To achieve this, we evaluated the expression of pro-inflammatory cytokines, including IL-6, IL-17, and TNF-α, in Jurkat cells.

### Interleukin analysis

#### Immunofluorescence assay

Immunofluorescence Assay (IF1088) for Getein1100 (Fluorescent chromatography “Getein1100” GETEIN Inc. China)) was used to determine the interleukin IL-6 was used to determine the interleukin IL-6 content in the supernatant of Jurkat cells incubated under different conditions.

In the test, an anti-human IL-6 monoclonal antibody I conjugated with fluorescence latex coated on the junction of the nitrocellulose membrane and the sample pad, and another anti-human IL-6 monoclonal antibody Ⅱ coated on the test line were used.

Upon application of the sample to the test strip, the fluorescence latex-labelled anti-human IL-6 antibody I specifically binds to the IL-6 present in the sample, resulting in the formation of a distinct antigen-antibody complex. This complex subsequently migrates to the detection zone of the test card via capillary action. At this point, the marked antigen-antibody complex is captured on the test line by a second antibody, anti-human IL-6 antibody II. The fluorescence intensity observed at the test line correlates positively with the concentration of IL-6 in the sample, thereby facilitating quantification through an immunofluorescence assay (refer to plate number 1 for Getein1100 methodology).

The test card is then inserted into the immunofluorescence quantitative analyser, the Getein1100. This device accurately measures the concentration of IL-6 in the sample, with the resulting value displayed on the screen. Additionally, the data is stored within the Getein1100 system and can be downloaded for further analysis or record-keeping purposes.

The obtained results can be readily integrated with the laboratory information system. The measurement range for IL-6 is established between 1.0 and 4,000.0 pg/mL, with a lower detection limit of ≤1.0 pg/mL. The reference range for IL-6 is identified as 7.0 pg/mL, determined through the normal distribution method at a 95% confidence interval.

#### Immunoenzymatic assay

An immunoenzymatic assay was used to quantify the levels of IL-17 (Human ELISA Kit from Abcam, USA 216167) and TNF-α (ELISA Kit from Immunodiagnostics K 9610, Germany) in the supernatant of Jurkat cells under various experimental conditions.

The ethical protocol for the study was approved by the Animal Ethical Committee of Tbilisi State Medical University (26.07.22).

### Statistical analysis

The Shapiro-Wilk test for normality was used to test the normality of the experimental data.

Analysis of Variance (Factorial ANOVA) was used to analyse the differences between the mean values of different experimental groups. Sample size per group (n = 5) and power (>80%) were *estimated* under the Fixed Effects model for significance level alpha <0.05 and RMSSE = 1,45. The RMSSE value was chosen based on typical values of the within-group standard deviation of the various characteristics in our preliminary studies and the requirement to reliably detect a difference of approximately 10%–15% between group means. The Tukey’s HSD test was used after analysis of variance (ANOVA) to determine which specific pairs of group means were significantly different from each other.

Statistical software SPSS-11 was used for data analysis and visualisation of results.

## Results

**FIGURE 1:**
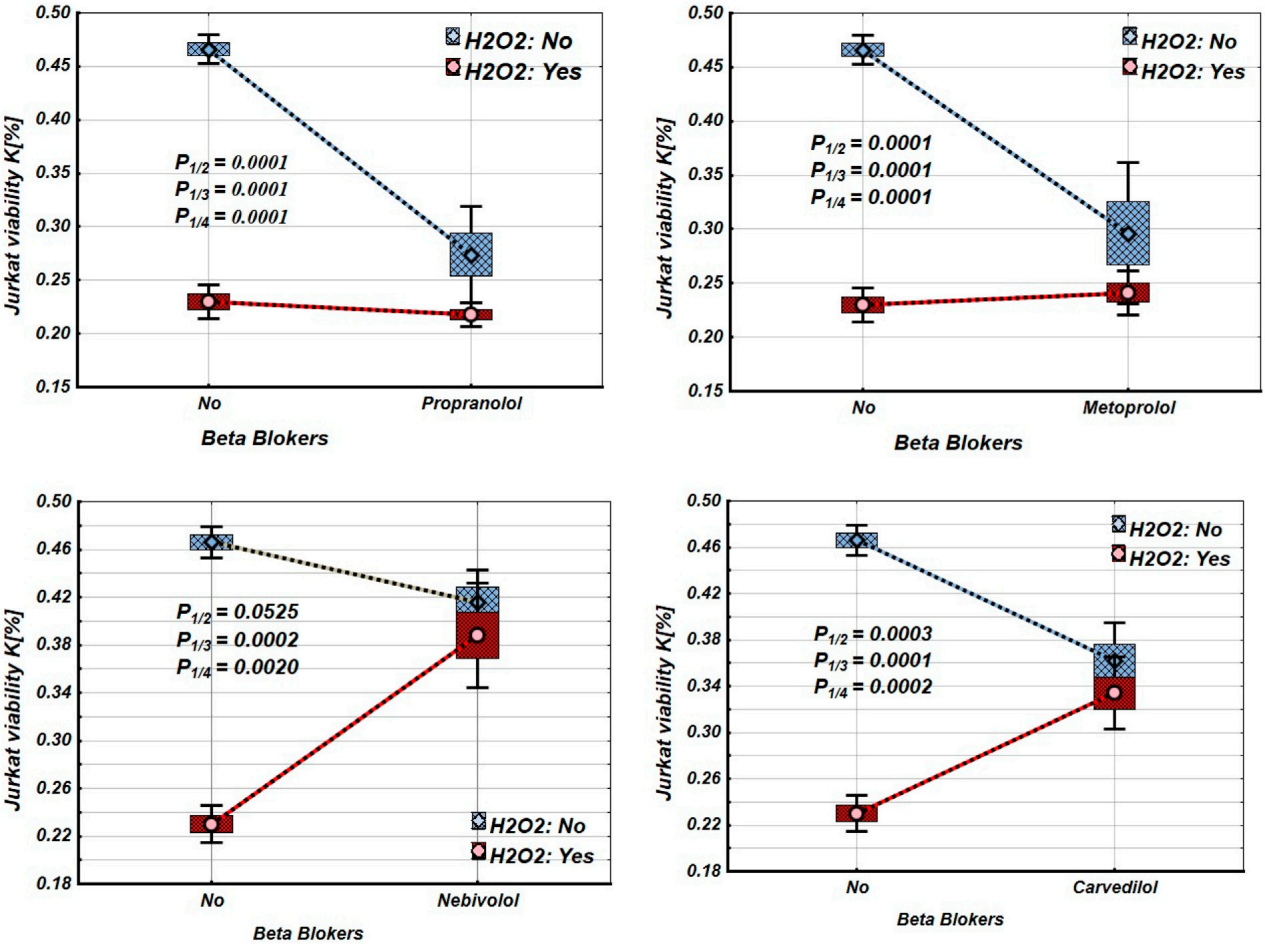
Effects of β-blockers on Mean values, Standard Errors and Standard Deviations of the Jurkat cell viability (intact (without H_2_O_2_) - blue line, and incubated under oxidative stress conditions (with H_2_O_2_ in concentration 50 µM) – red line). Analysis of Variance (Factorial ANOVA). P_1/2_ - statistical significance of the difference between the mean viability of intact Jurkat cells and those treated with different β-blockers; P_1/3_ - statistical significance of the difference in viability between intact Jurkat cells and those incubated under oxidative stress conditions (with H_2_O_2_). P_1/4_ - statistical significance of the difference in viability between intact Jurkat cells and those incubated under oxidative stress when treated with various β-blockers (Propranolol – 36 μg/mL, Carvedilol – 10 μg/mL, Nebivolol – 4 μg/mL, Metoprolol - 20 μg/mL) (Tukey HSD test; Jurkat cells viability. Approximate Probabilities for Post Hoc Tests).

The study results show that propranolol, metoprolol, and carvedilol significantly decreased the viability of intact Jurkat cells, but nebivolol did not (Figure 1; Table 1). Under incubation in oxidative stress conditions, the viability of Jurkat cells decreased significantly. Propranolol and metoprolol did not affect the viability of Jurkat cells incubated under oxidative stress conditions, whereas nebivolol and carvedilol improved the viability of Jurkat cells under these conditions (Figure 1).

**TABLE 1:** Mean values and statistical significance of the difference between the means of Jurkat cell viability under different β-blocker and H_2_O_2_ exposure conditions (N–without H_2_O_2_ and/or β-blockers; Y–with H_2_O_2_ and/or β-blockers). (Tukey HSD test; Jurkat cells viability. Approximate Probabilities for Post Hoc Tests).

Groups		β-blockers							
		Propranolol		Metoprolol		Nebivolol		Carvedilol	
H_2_O_2_	β-blockers	Mean	P	Mean		Mean	P	Mean	P
N	N	0.466		0.466		0.466		0.466	
N	Y	0.274	0.0001	0.296	0.0001	0.416	0.0525	0.362	0.0003
Y	N	0.230	0.0001	0.230	0.0001	0.230	0.0002	0.230	0.0001
Y	Y	0.218	0.0001	0.241	0.0001	0.388	0.002	0.334	0.0002

**FIGURE 2:**
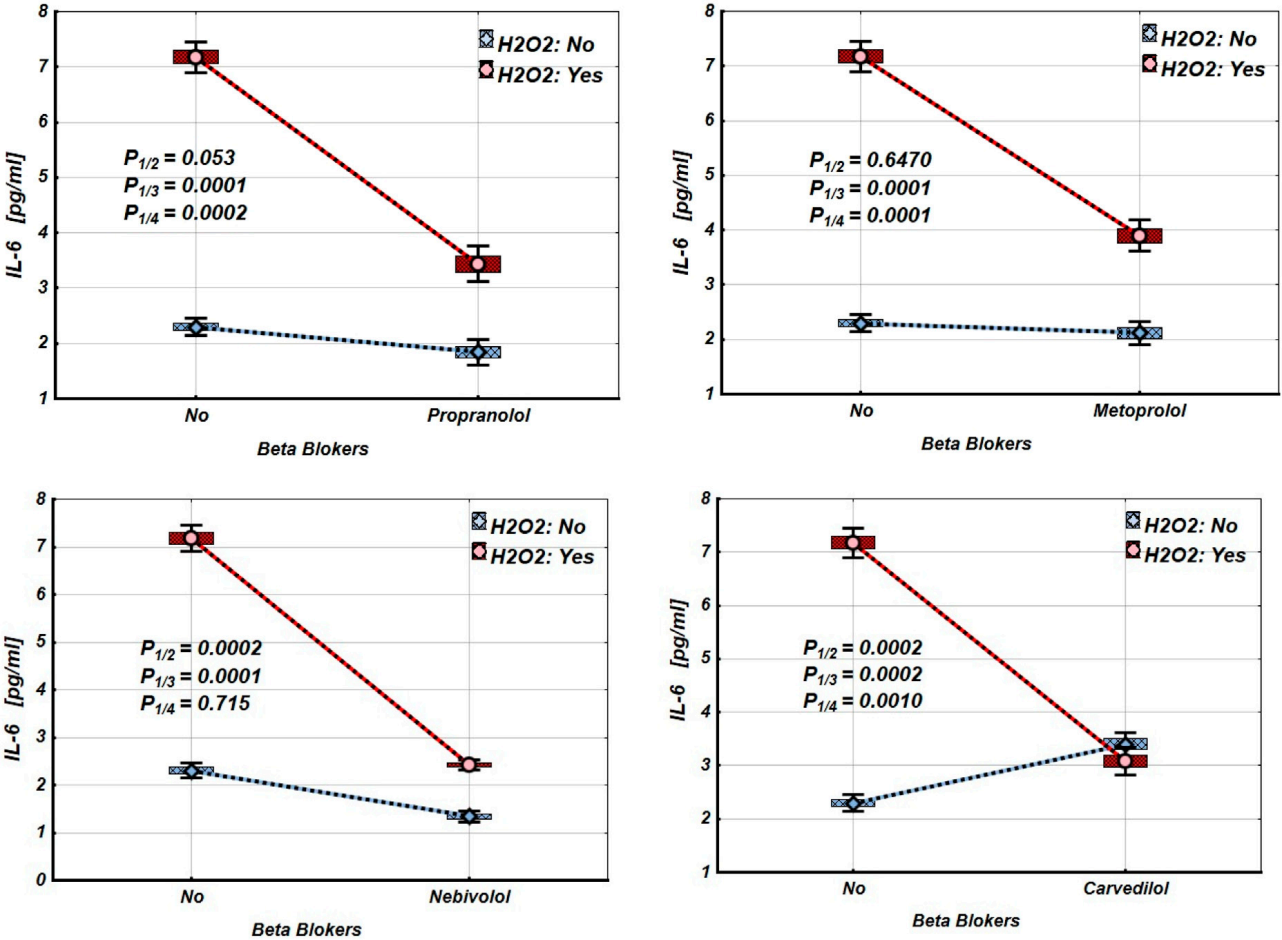
Impact of β-blockers on the Mean values, Standard Errors and Standard Deviations of IL-6 expression levels in Jurkat cells (intact (without H_2_O_2_) - blue line, and incubated under oxidative stress conditions (with H_2_O_2_ in concentration 50 µM) – red line). (Analysis of Variance (Factorial ANOVA)). P_1/2_ - statistical significance of the difference in IL-6 expression levels between the intact Jurkat cells and those treated with different β-blocker; P_1/3_ - statistical significance of the difference in IL-6 expression levels between intact Jurkat cells and those subjected to oxidative stress (with H_2_O_2_); P_1/4_ - statistical significance of the difference in IL-6 expression levels between intact Jurkat cells and those under oxidative stress with different β-blockers treatment (Propranolol – 36 μg/mL, Carvedilol – 10 μg/mL, Nebivolol – 4 μg/mL, Metoprolol - 20 μg/mL) (Tukey HSD test; IL-6 expression levels. Approximate Probabilities for Post Hoc Tests).

**FIGURE 3:**
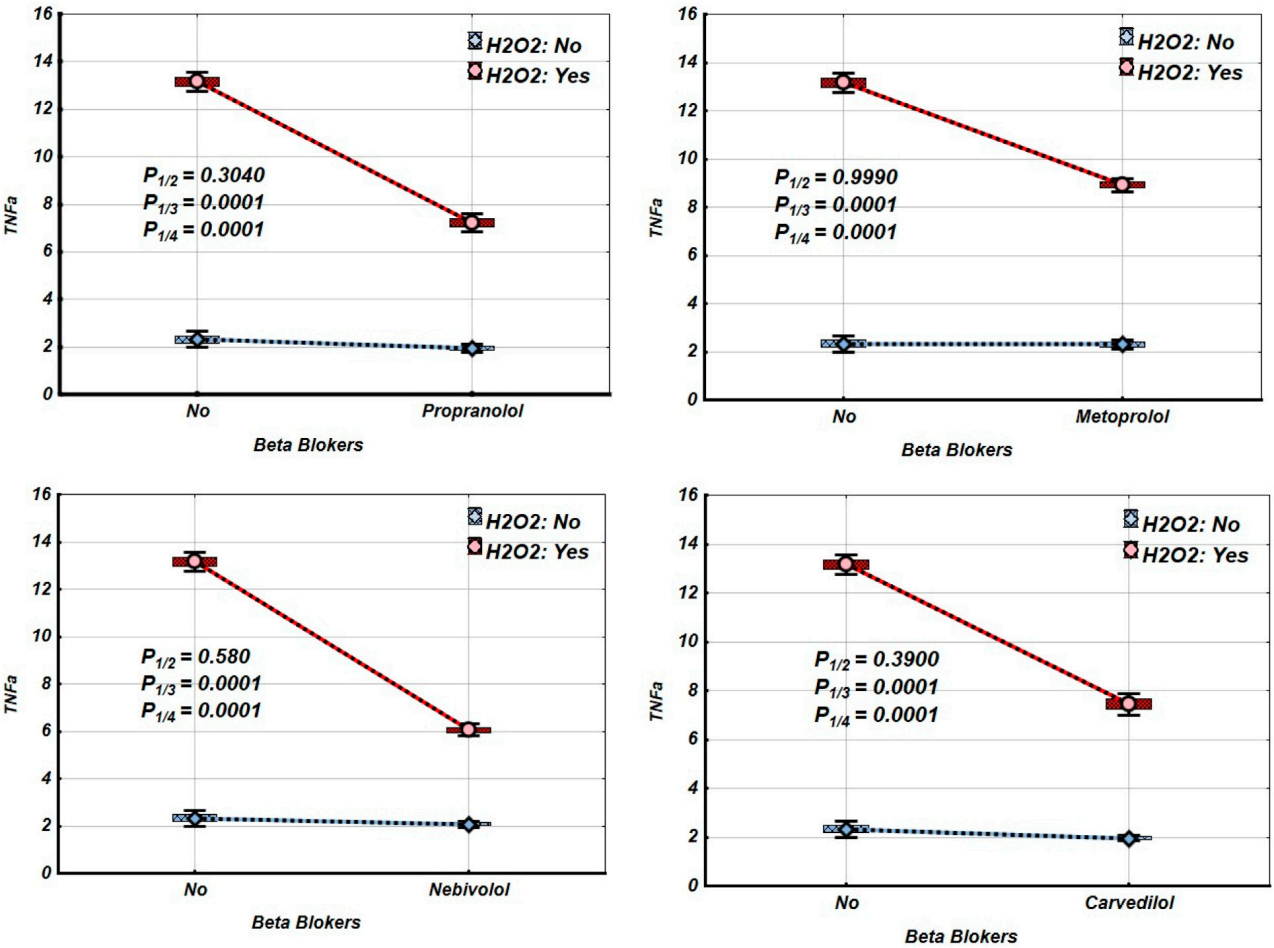
Impact of β-blockers on the Mean values, Standard Errors and Standard Deviations of TNF-α expression levels in Jurkat cells (intact (without H_2_O_2_) - blue line, and incubated under oxidative stress conditions (with H_2_O_2_ in concentration 50 µM) – red line). (Analysis of Variance (Factorial ANOVA)). P_1/2_ - statistical significance of the difference in TNF-α expression levels between the intact Jurkat cells and those treated with different β-blocker; P_1/3_ - statistical significance of the difference in TNF-α expression levels between intact Jurkat cells and those subjected to oxidative stress (with H_2_O_2_); P_1/4_ - statistical significance of the difference in TNF-α expression levels between intact Jurkat cells and those under oxidative stress with different β-blockers treatment (Propranolol – 36 μg/mL, Carvedilol – 10 μg/mL, Nebivolol – 4 μg/mL, Metoprolol - 20 μg/mL) (Tukey HSD test; TNF-α expression levels. Approximate Probabilities for Post Hoc Tests).

**FIGURE 4:**
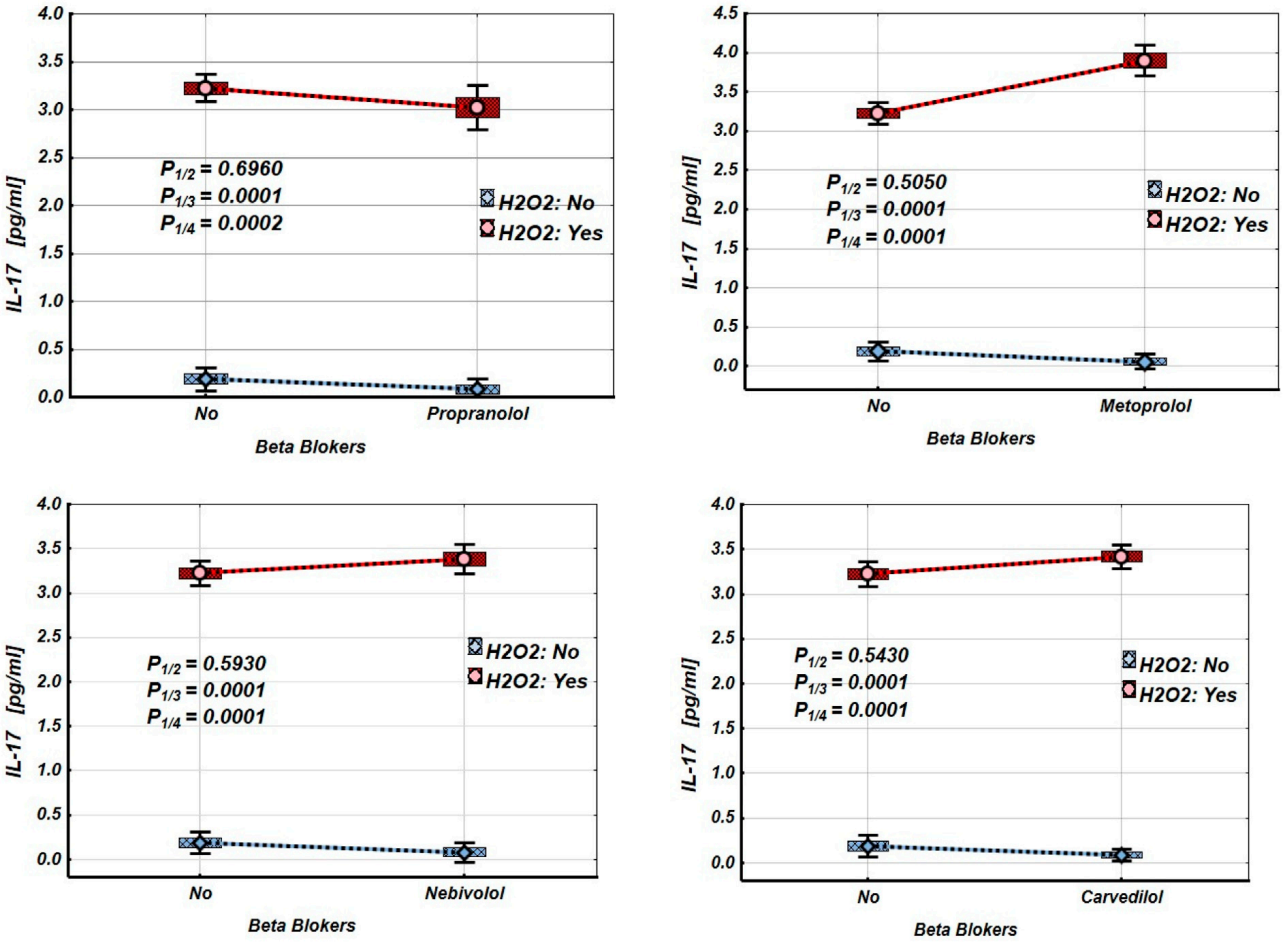
Impact of β-blockers on the Mean values, Standard Errors and Standard Deviations of IL-17 expression levels in Jurkat cells (intact (without H_2_O_2_) - blue line, and incubated under oxidative stress conditions (with H_2_O_2_ in concentration 50 µM) – red line). (Analysis of Variance (Factorial ANOVA)). P_1/2_ - statistical significance of the difference in IL-17 expression levels between the intact Jurkat cells and those treated with different β-blocker; P_1/3_ - statistical significance of the difference in IL-17 expression levels between intact Jurkat cells and those subjected to oxidative stress (with H_2_O_2_); P_1/4_ - statistical significance of the difference in IL-17 expression levels between intact Jurkat cells and those under oxidative stress with different β-blockers treatment (Propranolol – 36 μg/mL, Carvedilol – 10 μg/mL, Nebivolol – 4 μg/mL, Metoprolol - 20 μg/mL) (Tukey HSD test; IL-17 expression levels. Approximate Probabilities for Post Hoc Tests).

Figures 2–4 and Tables 2–4 illustrate the expression levels of IL-6, IL-17, and TNF-α in intact Jurkat cells and those incubated under oxidative stress conditions. It is evident that oxidative stress significantly increased the expression levels of these cytokines in Jurkat cells. Propranolol, metoprolol, nebivolol, and carvedilol showed no significant effects on the expression levels of IL-6, IL-17, and TNF-α in intact Jurkat cells (Figures 2–4 (blue line)). However, these β-blockers led to a notable reduction in the expression of IL-6 and TNF-α in Jurkat cells subjected to oxidative stress conditions (Figures 2, 3 (red line), Table 2, 3). Conversely, the levels of IL-17 remained largely unaffected by the presence of β-blockers (Figure 4 (red line), Table 4).

**TABLE 2:** Mean values and statistical significance of the difference between the means of the IL-6 expression levels in Jurkat cells under different β-blocker and H_2_O_2_ exposure conditions (N–without H_2_O_2_ and/or β-blockers; Y–with H_2_O_2_ and/or β-blockers). (Tukey HSD test; IL-6 expression levels. Approximate Probabilities for Post Hoc Tests).

Groups		β-blockers							
		Propranolol		Metoprolol		Nebivolol		Carvedilol	
H_2_O_2_	β-blockers	Mean	P	Mean		Mean	p	Mean	P
N	N	2.300		2.300		2.300		2.300	
N	Y	1.840	0.053	2.120	0.649	1.340	0.0002	3.400	0.0002
Y	N	7.180	0.0001	7.180	0.0001	7.180	0.0001	7.180	0.0002
Y	Y	3.440	0.0002	3.900	0.0001	2.420	0.715	3.080	0.0010

**TABLE 3:** Mean values and statistical significance of the difference between the means of the TNF-α expression levels in Jurkat cells under different β-blocker and H_2_O_2_ exposure conditions (N–without H_2_O_2_ and/or β-blockers; Y–with H_2_O_2_ and/or β-blockers). (Tukey HSD test; TNF-α expression levels. Approximate Probabilities for Post Hoc Tests).

Groups		β-blockers							
		Propranolol		Metoprolol		Nebivolol		Carvedilol	
H_2_O_2_	β-blockers	Mean	p	Mean		Mean	P	Mean	P
N	N	2.320		2.320		2.320		2.320	
N	Y	1.940	0.304	2.300	0.999	2.080	0.580	1.960	0.395
Y	N	13.160	0.0001	13.160	0.0001	13.160	0.0001	13.160	0.0001
Y	Y	7.220	0.0001	8.920	0.0001	6.060	0.0001	7.440	0.0001

**TABLE 4:** Mean values and statistical significance of the difference between the means of the IL-17 expression levels in Jurkat cells under different β-blocker and H_2_O_2_ exposure conditions (N–without H_2_O_2_ and/or β-blockers; Y–with H_2_O_2_ and/or β-blockers). (Tukey HSD test; IL-17 expression levels. Approximate Probabilities for Post Hoc Tests).

Groups		β-blockers							
		Propranolol		Metoprolol		Nebivolol		Carvedilol	
H2O2	β-blockers	Mean	p	Mean		Mean	p	Mean	p
N	N	0.188		0.188		0.188		0.188	
N	Y	0.080	0.696	0.060	0.505	1.340	0.593	0.087	0.543
Y	N	3.222	0.0001	3.222	0.0001	3.222	0.0001	3.222	0.0001
Y	Y	3.020	0.0002	3.900	0.0001	2.420	0.0001	3.418	0.0001

## Discussion

Jurkat cells, unlike primary human T cells, exhibit significant genetic and phenotypic stability, as well as high reproducibility across experiments. They effectively eliminate donor variability and remove the need for human subject approval or invasive procedures. These features make Jurkat cells an ideal choice for controlled pharmacological studies, particularly during the preclinical exploratory phase, where both practicality and ethical considerations are critical (Swiss Association of Research Ethics Committees, 2025).

Jurkat T cells are particularly responsive to oxidative stimuli, which play a significant role in various pathological conditions, including inflammation, autoimmune diseases, and hypertension. Inflammatory immune cells, like neutrophils and macrophages, release ROS, including hydrogen peroxide. Researchers often use exogenous H_2_O_2_ in laboratory studies to mimic the oxidative stress associated with chronic inflammation (Nathan and Ding, 2000; Mittal et al., 2014; Touyz and Briones, 2011). Extensive literature demonstrates that exposure to H_2_O_2_ exacerbates oxidative stress in Jurkat cells, as evidenced by increased lipid peroxidation (Rhee et al., 2012; Enukidze et al., 2009) and protein oxidation (Pace et al., 2025). This oxidative stress triggers a transcriptional response (Taylor et al., 2022) and is linked to apoptosis as well as cytokine production (Nindl et al., 2004). The application of H_2_O_2_ serves as a valuable tool for exploring redox-sensitive pathways and evaluating the effects of pharmacological agents, such as β-blockers, on cytokine production and cell viability within an inflammatory context (Los et al., 1995; Pelicano et al., 2004; Veal et al., 2007).

The activation of β-adrenergic receptors in T cells leads to an increase in intracellular levels of cyclic adenosine monophosphate (cAMP). This rise in cAMP activates protein kinase A (PKA), which subsequently modulates transcription factors such as nuclear factor kappa-light-chain-enhancer of activated B cells (NF-κB) and activator protein 1 (AP-1). This mechanism, in conjunction with the T cell receptor (TCR) signalling pathway, effectively regulates the expression of pro-inflammatory cytokines, including TNF-α, IL-2, and IL-6 (Lorton and Bellinger, 2015). Under specific stress conditions, enhanced β-adrenergic signalling can alter T cell activation profiles toward pro-inflammatory phenotypes, potentially contributing to immune dysregulation (Flierl et al., 2008; Mancia et al., 2022). Pharmacological inhibition of β-adrenergic receptors is a well-established therapeutic strategy for managing hypertension. However, it also has significant immunomodulatory effects. Research indicates that β-blockers can reduce cAMP/PKA signalling, decrease the activation of NF-κB and AP-1, and ultimately alter the cytokine secretion profiles in immune cells (Nance and Sanders, 2007).

Our experimental results demonstrated that propranolol, carvedilol, and metoprolol decreased the viability of Jurkat T cells, whereas nebivolol did not significantly impact cell viability (see Figure 1; Table 1). The cytotoxic effects of these β-blockers appear to be linked to the disruption of cAMP/PKA signalling, impairment of mitochondrial function (Balaban et al., 2005), and increased oxidative stress (Hajighasemi and Mirshafiey, 2009). Notably, metoprolol, due to its β_1_-selectivity, causes a relatively smaller reduction in PKA activity compared to propranolol (Wang et al., 1999; Sharashenidze et al., 2022), which corresponds to its milder effect on Jurkat cell survival. In contrast, carvedilol’s dual blockade of β-adrenergic and α_1_ receptors significantly affects cAMP signalling and calcium homeostasis, resulting in enhanced apoptosis in Jurkat cells (see Table 1 (Figure 1; Table 1). The viability of Jurkat cells significantly decreased when they were incubated under oxidative stress conditions (see Table 1; Figure 2). This decline is likely linked to the activation of several cell death mechanisms, including mitochondrial dysfunction, DNA damage, and apoptosis, which is mediated by the Bcl-2 and Caspase-8 pathways (Chiaramonte et al., 2001). Furthermore, the upregulation of interferon-gamma (IFN-γ) release seems to promote the expression of RNA-dependent protein kinase (PKR) (Pyo et al., 2008).

According to our study results, nebivolol, carvedilol, metoprolol, and propranolol improved the viability of Jurkat cells under oxidative stress, with nebivolol showing the most pronounced effect. This finding aligns with previous reports suggesting that certain β-blockers have antioxidant properties, help modulate redox balance, and influence mitochondrial stability (Rashida Gnanaprakasam et al., 2018; Celik et al., 2016; Ricci et al., 2023). These results indicate that specific β-blockers may have therapeutic potential by enhancing cellular resilience against oxidative damage. Furthermore, the varying effects of these β-blockers on Jurkat T-cell survival highlight the need for careful interpretation and consideration in future therapeutic strategies.

Nebivolol exhibits a strong cytoprotective effect, which aligns with its unique pharmacological characteristics. Unlike traditional β_1_-selective or non-selective β-blockers, nebivolol has vasodilatory and antioxidative properties. These effects are partly mediated by the activation of β_3_-adrenergic receptors, which leads to the phosphorylation of endothelial nitric oxide synthase (eNOS) in endothelial cells (Chlopicki et al., 2002; de Boer et al., 2007). In studies of vascular and myocardial models, nebivolol has been shown to inhibit NADPH oxidase activity, reduce the generation of superoxide, and maintain the availability of nitric oxide (NO). This helps mitigate oxidative stress and protects mitochondrial function (Celik et al., 2016; Oelze et al., 2006). However, these mechanisms have not been tested directly in Jurkat cells, which do not have β_3_-adrenergic receptor-mediated eNOS signalling like endothelial cells do. Instead, Jurkat cells can produce NO through inducible nitric oxide synthase (iNOS), which plays a role in mitochondrial regulation and Fas-mediated apoptosis (Förstermann and Sessa, 2012). This suggests that nebivolol may indirectly influence NO-dependent pathways in lymphocytes under oxidative stress, although this hypothesis requires further validation through direct measurements of intracellular NO.

Another plausible explanation for the protective effect of nebivolol relates to its impact on ROS homeostasis. Research has shown that nebivolol can suppress oxidative bursts in inflammatory cells, which is consistent with the inhibition of NADPH oxidase-dependent ROS production (Celik et al., 2016). Furthermore, studies conducted in non-immune systems have demonstrated that nebivolol stabilises membrane potential and limits oxidative injury to mitochondria, thereby enhancing cell survival (Chlopicki et al., 2002). Taken together, these data suggest that nebivolol may exert multifactorial protective effects against oxidative stress, including both ROS suppression and NO-related signalling pathways, although confirmation of this in Jurkat cells is still necessary.

Carvedilol, a non-selective β-blocker with α-adrenergic antagonistic properties, demonstrated significant cytoprotective effects. This aligns with its well-documented antioxidative properties, which include direct scavenging of ROS, inhibition of lipid peroxidation, and suppression of NF-κB activity. These effects have been observed in both cardiovascular and immune-related contexts (Chen-Scarabelli et al., 2012).

In contrast, metoprolol and propranolol demonstrated more limited protective effects in Jurkat cells. This is likely due to their weaker intrinsic antioxidant capacity and a less significant impact on mitochondrial stability (Bourgonje et al., 2018).

Our findings suggest that β-blockers vary in their ability to protect Jurkat T-cells from oxidative stress, with nebivolol and carvedilol demonstrating the most significant effects. The protective mechanisms likely involve redox-sensitive pathways, which may include the suppression of reactive oxygen species (ROS) and stabilisation of mitochondria. Additionally, it is plausible that these mechanisms are linked to nitric oxide (NO) signalling. However, these proposed mechanisms in T-cells remain speculative, and further investigations will be needed to confirm their role in protecting immune cells. Differences in protective efficacy highlight the pharmacological diversity among β-blockers, indicating that carvedilol and nebivolol may offer specific advantages in scenarios where oxidative stress contributes to immune dysfunction.

Jurkat cells, which serve as an immature T-cell model, typically exhibit limited cytokine production when unstimulated. Under normal conditions, they release only low to moderate levels of IL-6 and TNF-α. These cytokines are essential mediators of immune responses, acute-phase reactions, and the regulation of inflammation. Effective cytokine production requires specific stimuli, such as oxidative stress, mitogens like phorbol 12-myristate 13-acetate (PMA) and ionomycin, or TCR activation using anti-CD3 and anti-CD28 antibodies (Khalaf et al., 2010; Radom-Aizik S, et al., 2007; Smeets et al., 2012).

The induction of cytokine production is controlled by well-characterised signalling pathways, including the NF-κB pathway, mitogen-activated protein kinase (MAPK) pathways, c-Jun N-terminal kinase (JNK) pathway, and extracellular signal-regulated kinase (ERK) pathway. These canonical cascades are crucial for converting external signals into appropriate cellular responses, forming part of the complex network that regulates immune activity. These pathways activate transcription factors such as AP-1, CCAAT/enhancer-binding protein beta (C/EBPβ), and signal transducer and activator of transcription 3 (STAT3), which in turn promote the expression of pro-inflammatory genes (Napetschnig and Wu, 2013; Liu et al., 2017; Karin, 2006).

Oxidative stress enhances the activation of NF-κB and MAPKs, which in turn activate the IL-6 promoter (Karin, 2006; Kishimoto, 2010; Dinarello, 2000). After IL-6 is secreted, it binds to its receptor (IL-6R), initiating the Janus kinase/Signal Transducer and Activator of Transcription 3 (JAK/STAT3) signalling pathway. This process establishes a positive autocrine feedback loop that sustains IL-6 expression and the inflammatory response (Alghamdi and Alissa, 2025).

Similarly, under oxidative stress, the production of TNF-α is increased via NF-κB. Once secreted, TNF-α activates its receptors (TNFR1/2), triggering the NF-κB, MAPK, and JAK/STAT cascades, which help maintain its own expression (Nakamura et al., 2011).

In contrast, IL-17 is primarily secreted by Th17 cells, while Jurkat cells have a limited ability to differentiate into Th17-like cells. As a result, IL-17 production in these cells remains modest, even under oxidative stress.

Nevertheless, oxidative stress can activate NF-κB, STAT3, RORγt, and MAPKs, all of which collectively contribute to IL-17 induction (Park et al., 2014; Zhao et al., 2023). NF-κB facilitates the nuclear translocation and promoter binding of p65, *while RORγt drives Th17 lineage-specific transcription.* The cooperation between RORγt, NF-κB, and STAT3 amplifies IL-17 production, which is further supported by IL-6-mediated STAT3 activation (Mangan et al., 2006; Huangfu et al., 2023).

Beta-blockers can modulate immune responses, including the production of cytokines, and suppress the activation of inflammatory pathways in various immune cell types (Bhol et al., 2024; Landegren et al., 1985). However, the specific effects of β-blockers on cytokine expression in Jurkat T lymphocytes have not been thoroughly investigated compared to their well-documented effects in primary immune cells. Our study aimed to investigate the effects of propranolol, metoprolol, nebivolol, and carvedilol on the secretion of IL-6, IL-17, and TNF-α by Jurkat cells under oxidative stress conditions.

Our findings show that exposing Jurkat cells to oxidative stress significantly increases the expression of IL-6, TNF-α, and IL-17, as summarised in Tables 2–4 and Figures 2–4. Interestingly, propranolol, metoprolol, nebivolol, and carvedilol did not significantly alter cytokine production in non-stimulated (intact) Jurkat cells. However, all four β-blockers exhibited a strong inhibitory effect on the release of IL-6 and TNF-α in the presence of oxidative stress. In contrast, the secretion of IL-17 was not significantly affected by β-blocker treatment in our study, even under oxidative stress conditions (Figure 4; Table 4).

**FIGURE 5:**
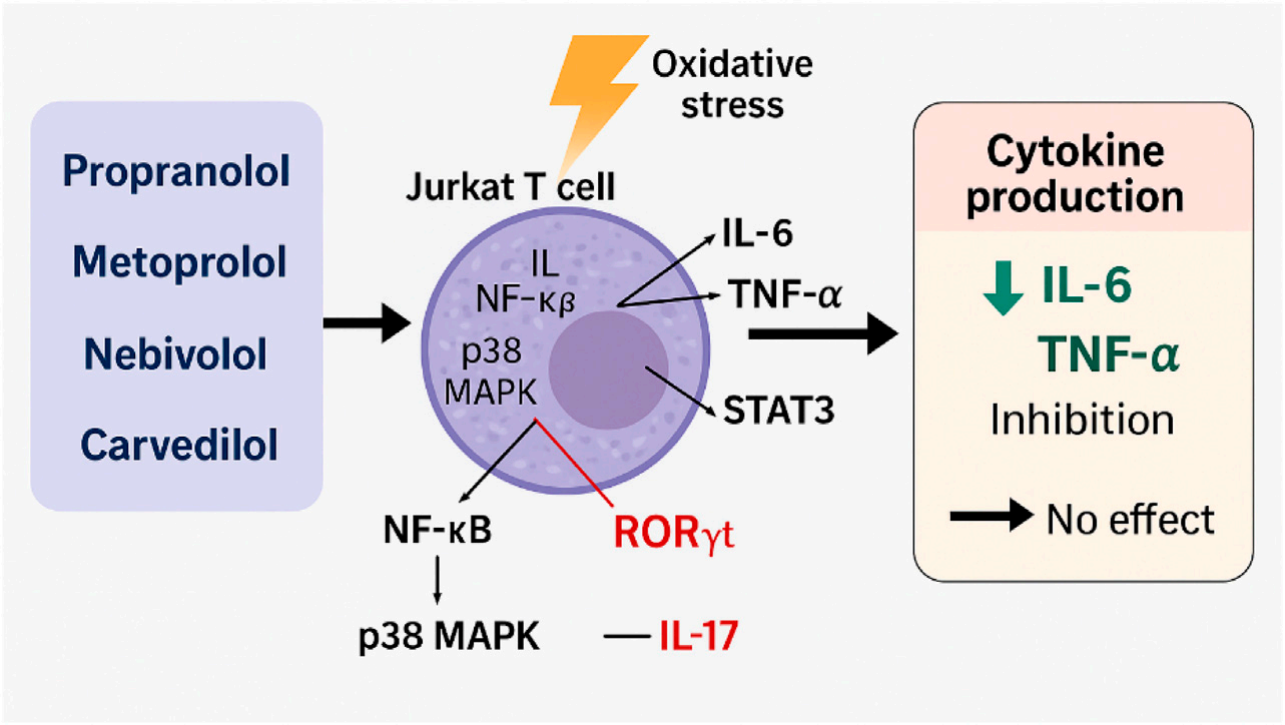
Modulation of cytokine production in Jurkat T cells under oxidative stress conditions.

The significant reducing effect of non-selective β1/β2-adrenergic receptor antagonists, such as propranolol, on L-6 and TNF-α secretion in stressed Jurkat cells is primarily due to the inhibition of β_2_-adrenergic receptor signalling, which plays a crucial role in modulating inflammatory responses (Chhatar and Lal, 2021). Notably, previous studies have shown that propranolol decreases the levels of IL-2, IL-10, and IFN-γ in phytohemagglutinin (PHA)-stimulated Jurkat cells, while having no such effect in unstimulated cells. This observation suggests that inhibitory effects of propranolol are related to target modulation of signalling pathways rather than to cytotoxicity (Lomsadze et al., 2013; Hajighasemi and Mirshafiey, 2016; Sharashenidze et al., 2021). Furthermore, propranolol has been shown to suppress NF-κB and interfere with p38 MAPK signalling. These mechanisms likely explain its selective inhibition of IL-6 and TNF-α in contrast to the production of IL-17, which appears to be regulated via the STAT3–RORγt axis rather than NF-κB-dependent pathways (Pantziarka et al., 2016; Qiao et al., 2018; Hiller et al., 2020; Sprague et al., 2021; Ağaç et al., 2018) (Figure 5).

Metoprolol, a β1-selective adrenergic antagonist, has shown a weaker ability to modulate cytokine activity compared to non-selective β-blockers. This difference can be attributed to the limited role of β1-adrenergic receptors in regulating T-cell cytokines (Lorton and Bellinger, 2015). The literature rarely reports on metoprolol’s direct inhibition of p38 MAPK; its modest effects on IL-6 and TNF-α are likely due to indirect modulation of NF-κB and translational control processes that do not affect mRNA expression (Calò et al., 2005; Ulleryd et al., 2014; Ricci et al., 2023; Yang et al., 2023). This helps explain why metoprolol is less effective compared to propranolol and nebivolol (Drygała et al., 2024) (Figure 5).

Nebivolol, compared to propranolol and metoprolol, shows a unique anti-inflammatory profile and the broadest cytokine-blocking activity. This is attributed to its dual mechanism of action: β1-selective antagonism and NO-mediated modulation of NF-κB. While the specific effects of nebivolol on NO release and its signalling pathways in T-cells are not fully understood, it is known that T-lymphocytes respond to NO donors. These donors affect TCR-mediated cytokine secretion through both cGMP-dependent and cGMP-independent pathways (Niedbala et al., 2006). This observation suggests that nebivolol reduces the oxidative modification of induced nitric oxide (iNO), improves its bioavailability, and can inhibit NF-κB through nitric oxide-dependent mechanisms observed in other cell types. Nebivolol achieves this inhibition by preventing the degradation of IκBα, which leads to a subsequent reduction in the transcription of NF-κB-dependent cytokines, such as IL-6 and TNF-α (Reynaert et al., 2004; Marshall et al., 2004).

This mechanism may explain the observed downregulation of IL-6 and TNF-α by nebivolol in our model, while its effects on IL-17 remain minimal. The expression of IL-17 is more closely associated with the activity of STAT3/RORγt rather than NO-dependent signalling. However, due to the lack of direct evidence, this hypothesis remains speculative, underscoring the need for experimental validation in Jurkat or primary T-cell models.

Carvedilol stands out among β-blockers because of its significant antioxidant and anti-inflammatory properties. Research indicates that carvedilol inhibits the nuclear translocation of NF-κB in T cells and macrophages, leading to a reduction in the secretion of pro-inflammatory cytokines such as IL-6 and TNF-α. Notably, carvedilol has a minimal effect on IL-17, which is primarily regulated by T-cell differentiation pathways, including the STAT3/RORγt signalling cascade. Unlike NF-κB, these pathways are less significantly affected by oxidative stress, emphasising the unique therapeutic potential of carvedilol in modulating inflammatory responses (Yang et al., 2003; Calò et al., 2005).

Under physiological conditions, catecholamines released during sympathetic activation bind to β-adrenergic receptors on lymphocytes, macrophages, and other immune cells. This interaction enhances the release of pro-inflammatory cytokines through the NF-κB and MAPK pathways. *In vivo*, β-blockers disrupt catecholamine-driven signaling and indirectly reduce cytokine production by dampening the adrenergic activation of immune cells initiated by the sympathetic nervous system (Yang et al., 2023; Ulleryd et al., 2014; Hajiaghayi et al., 2024). However, our Jurkat cell model, which does not have its own catecholamine influx, does not fully capture the complexity of adrenergic regulation found in primary immune cells or *in vivo* systems. This model represents a simplified system where oxidative stress triggers cytokine production. Consequently, the consistent suppression of IL-6 and TNF-α production in our Jurkat culture system under oxidative stress, by propranolol, metoprolol, nebivolol, and carvedilol, is likely related to their direct redox-modulatory properties (such as antioxidant activity and NO-mediated signalling) and their capacity to inhibit intracellular inflammatory pathways (NF-κB and p38 MAPK), rather than through modulation of adrenergic activation as observed *in vivo*. In contrast, the IL-17, whose induction is more dependent on STAT3 and RORγt, is relatively insensitive to β-blocker treatment.

Our results highlight that β-blockers vary significantly in their ability to modulate the immune response. Nebivolol and carvedilol provide the most substantial protection due to their combined actions as antioxidants, mitochondrial stabilisers, and suppressors of NF-κB. Propranolol and metoprolol have weaker, yet still notable effects. This pharmacological diversity suggests that certain β-blockers could effectively influence redox-sensitive cytokine responses, although further confirmation using primary T-cell models is necessary.

## Conclusion

This study examined the effects of β-blockers—specifically propranolol, metoprolol, nebivolol, and carvedilol—on cytokine production in Jurkat T lymphocytes under conditions of oxidative stress. The results showed that oxidative stress triggered the expression of cytokines IL-6, TNF-α, and IL-17 in the Jurkat culture system, indicating their sensitivity to redox imbalance. This reflects the intrinsic ability of cells to modulate inflammatory signalling. The effects of β-blockers appear to originate from direct mechanisms within the cells, such as antioxidant activity, nitric oxide-related signalling, and the suppression of intracellular inflammatory pathways like NF-κB and p38 MAPK. However, it should be noted that these *in vitro* findings do not fully capture the complex adrenergic regulation observed in primary immune cells *in vivo*.

The findings of this study highlight the differing impacts of β-blockers on redox-sensitive cytokine pathways. These medications exhibit varying levels of cytotoxic potential and anti-inflammatory effectiveness, which depend on their receptor selectivity and intrinsic antioxidant properties. This variability underscores their therapeutic immunomodulatory potential, particularly in situations involving oxidative stress. All four β-blockers tested were found to inhibit the release of IL-6 and TNF-α under oxidative stress conditions; however, none had a significant effect on IL-17 production. This outcome aligns with the understanding that IL-17 is primarily regulated by the STAT3/RORγt pathway rather than by the NF-κB/MAPK pathways.

Among the β-blockers tested, nebivolol and carvedilol showed the strongest cytoprotective and anti-inflammatory effects. These medications improved the viability of Jurkat cells and suppressed the expression of pro-inflammatory cytokines. This suggests that they may help alleviate inflammation-related dysfunction, likely due to their combined roles as antioxidants, mitochondrial stabilizers, and anti-inflammatory agents. This action is mediated through the NF-κB and MAPK signaling pathways. In the case of nebivolol, its effects are also related to nitric oxide signaling.

In contrast, metoprolol and propranolol exhibited more limited protective effects, which corresponded to their weaker intrinsic antioxidant capacities and reduced impact on the NF-κB/MAPK pathways.

Our findings provide strong evidence that certain β-blockers, particularly third-generation agents like nebivolol and carvedilol, offer dual benefits in managing hypertension. These medications appear to help maintain the viability of immune cells while simultaneously reducing the production of inflammatory cytokines.

To gain a better understanding of the immunoregulatory mechanisms of β-blockers and to enhance their clinical use in inflammation-related cardiovascular diseases, further research using primary immune cells and *in vivo* models is essential.

### Limitation

This study has several limitations that should be acknowledged:

- Jurkat T lymphocytes are commonly used to study T-cell signalling and cytokine responses; however, they do not fully replicate the functional and phenotypic complexity of primary human T lymphocytes. Due to their transformed nature, variations in receptor expression, redox sensitivity, and cytokine regulation may influence their response to beta-blockers and oxidative stimuli.
- Although the Jurkat model was selected for its biological relevance to T-cell-mediated immune responses and its suitability for initial mechanistic screening, it lacks the complex tissue microenvironment and cell–cell interactions present *in vivo*. Therefore, the observed effects of beta-blockers should be interpreted as direct, cell-intrinsic actions (e.g., antioxidant activity and suppression of NF-κB/MAPK) rather than as indirect modulation of adrenergic activation that occurs under physiological conditions.
- We did not directly assess the activation of NF-κB or p38 MAPK, nor did we quantify intracellular nitric oxide levels. Therefore, our interpretations concerning the signaling pathways influenced by β-blockers remain speculative and require confirmation through targeted molecular assays.

Our analysis focused on three cytokines: IL-6, TNF-α, and IL-17. We did not examine other cytokines that are relevant to β-adrenergic and T-cell signalling.

The concentrations of β-blockers used in this study (measured in µg/mL) are significantly higher than the plasma levels typically found in patients taking therapeutic oral doses (measured in ng/mL). It is important to emphasise that the *in vitro* exposures should be considered supra-physiological. The selected pharmacological ranges were deliberately chosen to elicit measurable mechanistic responses in cultured cells, rather than to precisely replicate clinical plasma concentrations.

In summary, while our results provide valuable insights into the different immunomodulatory effects of β-blockers under oxidative stress, future studies should employ physiologically relevant concentrations, use primary immune cells, conduct pathway-specific analyses, and incorporate *in vivo* models to validate and extend these observations.

## Data Availability

The raw data supporting the conclusions of this article will be made available by the authors, without undue reservation.
